# Experiences and attitudes of clinical and academic nurses about infection prevention and control nursing curriculum and duties - a qualitative study

**DOI:** 10.1186/s12909-024-05633-6

**Published:** 2024-06-17

**Authors:** Zahra Gorjian, Marziyeh Asadizaker, Kourosh Zarea, Alireza Irajpour, Fatemeh Ahmadi, Dariush Rokhafroz

**Affiliations:** 1 Student Research Committee, School of Nursing and Midwifery, Ahvaz Jundishapur University of Medical Sciences, Ahvaz, Iran, Abadan University of Medical Sciences, Abadan, Iran; 2 Nursing Care Research Center in Chronic Diseases, School of Nursing and Midwifery, Ahvaz Jundishapur University of Medical Sciences, Ahvaz, Iran; 3 Nursing & Midwifery Care Research Center, Isfahan University of Medical Sciences, Isfahan, Iran; 4 Infection and Tropical Disease Research Center, Health research institute, Ahvaz Jundishapur University of Medical Sciences, Ahvaz, Iran

**Keywords:** Nurse specialists, Communicable diseases, Prevention and control, Curriculum

## Abstract

**Background:**

Infectious diseases are becoming more widespread and re-emerging, causing psychological, social, economic, and health effects at both national and international levels. Specialist nurses can help prevent and control these infections. However, in Iran, there are currently no specialist infection prevention and control (IPC) nurses to manage and control infections. This study aims to explore clinical and academic nurses’ attitudes toward IPC nursing curriculum and duties.

**Methods:**

The study used a qualitative content analysis approach. Thirty-six participants, including clinical and academic nurses, were selected using a purposeful sampling method. Data was collected through seven focused group discussions. The accuracy and validity of the research tools were measured using the Four-Dimension Criteria developed by Lincoln and Guba. Data analysis was conducted using directed content analysis.

**Results:**

The data analysis of the discussions held in the seven focus groups extracted 628 codes. Three themes were developed from the qualitative analysis: “Core characteristics of the curriculum”, “Expected competencies and skills”, and “Evaluation.” These themes were derived from nine main categories and 25 subcategories.

**Conclusions:**

Specialist IPC nurses can play important roles in various positions and environments. Therefore, educational policymakers in Iran should consider establishing IPC nursing courses. It is also recommended that policymakers and decision-makers in the nursing field of other less developed countries should prioritize this issue.

## Introduction

Emerging and re-emerging infectious diseases have been a significant burden on the medical system in countries like Iran, particularly nosocomial infections. This burden causes adverse effects on disease treatment and leads to an increase in fatalities [1]. Infectious diseases also hinder social and economic development, making them a significant threat to international health security. The ongoing COVID-19 pandemic is a prime example of this. With the emergence of new diseases, increasing microbial resistance, international travel, and the risk of bioterrorism, infectious diseases have become a global concern that requires international and interdisciplinary coordination [2] Infection prevention and control (IPC) is a multidisciplinary educational program that provides specialized staff training and is necessary to improve public health [3].

Nurses are a vital part of healthcare systems and make up the largest group of healthcare workers (HCWs) [4]. They are often considered the backbone of healthcare systems as they spend more time with patients and can play a significant role in preventing and controlling infectious diseases. Therefore, it is essential for nurses to acquire the necessary knowledge, skills, and attitudes related to IPC [5].

A review of 16 studies on the number of nurses during the COVID-19 pandemic revealed that those with lower levels of professional education experienced increased burnout [6]. Professional and academic training has been found to be effective in increasing nurses’ preparedness and awareness of infection [7]. Nowadays, there are various professional courses available for nurses to enhance their knowledge and skills. A professional nurse is someone who has attained special knowledge at the Master of Science (MS) level and can make decisions with clinical and comprehensive qualifications [8]. The value of clinical nurses lies in their cost-effectiveness, which enables them to provide stable care to patients [9]. Studies in the US estimate that the use of professional nurses can lead to an annual savings of $8.75 [10]. Patients are more satisfied with the care provided by professional nurses than non-professional ones. Additionally, their organizational behavior and psychological reflections are positively evaluated [11]. There are increasing job opportunities for professional nurses, who have received extensive training in pharmacology, physiology, and physical examination as compared to special nursing courses [12].

Prevention and control are important areas of study for nurses, particularly in the field of IPC. IPC nurses are qualified to use effective methods to prevent the spread of infections and ensure the safe management of patients with infectious diseases [13]. They can educate healthcare professionals about the risk of hospital-acquired infections and advise them on how to reduce these risks. They also investigate the prevalence of diseases among patients affected by hospital diseases and work with patients to control these diseases [14]. Additionally, they study the occurrence of hospital-acquired infections among patients with weakened immune systems, chronic illnesses, and infants born with complications [15]. It is the responsibility of these nurses to analyze reports of infectious diseases and recommend innovative trends and policies to control infections in medical centers [16]. They also play a crucial role in planning prevention and control measures for epidemic infections in hospitals, based on evidence [17].

IPC nurses have a crucial responsibility of educating people on infection prevention and management, and also making them aware of the severe consequences that could occur [18]. By supporting hospital staff, nurses, and physicians, they help to minimize the risk of infections among patients, staff and visitors [19]. This is a challenging task for nurses who care for patients with infectious diseases, as they are also responsible for routine activities. However, professional IPC nurses can help prevent infections by focusing on this issue [20]. IPC nurses provide clients with leadership and specialist advice, as well as healthcare and social care organizations. They enable IPC services to provide evidence-based, timely, safe, adequate, and effective knowledge, expert advice, and support to the medical staff and other employees of the medical organization [21].

As previously mentioned, IPC nurses have a crucial role in training, research, care, management, and consultation. However, there are no established IPC nursing courses in Iran, and each hospital only has one IPC nurse who has completed a general nursing course without specialized academic education [22]. While developed countries like the US, Canada, and Australia have standardized infection control processes and resource management, there is a lack of leadership and management structures, planning, staff management, managing patients, source management, education, and focal consequences in Iran. This poses a significant challenge for health centers [23].

Nurses can play a vital role in preventing and controlling infectious diseases, but they lack the necessary academic skills in this area [24]. Therefore, it is crucial to design appropriate curricula for training nurses. The curriculum design should take into account the viewpoints of nurses, which are useful for medical stakeholders. A qualitative approach can provide a deep understanding of nurses’ perspectives who work in infectious wards, particularly during the COVID-19 pandemic. Their invaluable information on the required skills, roles, and places necessary for an IPC nurse can help researchers plan an appropriate curriculum to train IPC nurses. Thus, this qualitative study aims to collect nurses’ viewpoints on the necessity of IPC nurses. Since there is a lack of infection control curriculum in Iran, and insufficient information, this stage of the research aims to assess the needs of the curriculum beneficiaries.

## Methods

### Study design and setting

This qualitative content analysis study with a conventional approach was conducted in Ahvaz, Iran in 2023 and approved with the ethics code No.IR.AJUMS.REC.1400.303.

### Participants (sampling)

The sampling process for this study was conducted purposefully. The participants included clinical and academic faculty members, nursing supervisors, clinical nurses from infectious and COVID-19 departments, infection control nurses, and senior year nursing students. Before the study began, the researcher spoke with the participants in person and explained the reasons for conducting the research. The inclusion criteria for participation in the study were having consent, experience in teaching prevention and control of infection for faculty members, and work experience during the COVID-19 pandemic for nurses and nursing students. If participants showed withdrawal or reluctance to participate in the study, they were excluded.

Upon completion of the sampling process, the study included a total of six educational faculty (nursing), five clinical faculty (nursing), three infection control nurses, five supervisors, three head nurses, five nurses, and nine nursing students.

### Data collection

Data was collected between May and October 2022 through semi-structured questioning during focus group discussions. These questions were obtained from another study that compares infection prevention and control master’s programs in prestigious universities around the world. This study was conducted by the same research team and is currently in the printing stage. Existing curricula were examined, and it was found that they had common components such as course name and definition, mission and vision, objectives, teaching methods, expected characteristics of graduates, competencies/skills, and evaluation. These components were used to initiate discussions in focus groups with questions in these areas. Also, these findings provided the authors with a framework for data analysis in the directed content analysis method.

The participants were provided with information about the study’s purpose, and the details of the sessions including the location, time, and conditions were communicated to them via email. The participants voluntarily gave their informed consent, and their anonymity was strictly maintained while their privacy was respected to ensure that their viewpoints remained confidential.

Seven focus groups were arranged in the workplaces of the participants where they discussed their experiences and perspectives on IPC specialist nurses in a face-to-face discussion. For the participants who could not come to the discussion place, a virtual link was sent to participate in the discussion. The study aimed to explore the themes of the study through the participants’ viewpoints.

The focus groups were exploratory in nature, and the facilitator had no bias or interest in the research topic. The questions were designed according to the discussion guide. The starting point of each session was the question, “What is your idea regarding the importance of IPC nurses according to your experiences?” Throughout the focus group discussions, various exploratory questions were asked such as “What qualified competencies and skills should an IPC nurse have?” “How can IPC nurses be effective in various medical places?” and “What roles can an IPC nurse play?”

Each focus group session lasted from 75 to 90 min, and their voices were recorded via an MP3 and then transcribed. For the protection of their privacy, the participants’ names were coded.

### Analyzing data

The data in this study was analyzed using the Elo and Kyngäs approach, with data gathering and analysis conducted via directed content analysis. During the analysis phase, the researchers transcribed the discussions and read them multiple times to gain an overall understanding. As qualitative research requires data immersion, the researchers listened to the participants’ views several times. They then wrote an abstract for each interview session and extracted the exact meanings, underlining important phrases, identifying units of conceptions, and extracting initial codes. Three coders coded the data. similar codes placed in the Predefined categories. The MAXQDA 2020 software was used for data analysis and categorization.

To ensure the trustworthiness of the collected data, the researchers used Lincoln and Guba’s criteria. They engaged in data collection to determine the credibility of the collected data. Furthermore, the codes and categories were continuously investigated and reviewed by the authors to ensure dependability [25]. Two external observers monitored the processes of data analysis. Confirmability of all research processes, particularly data collection and analysis, as well as the formation of the main categories were approved by other external observers. Finally, maximum diversity was observed in the recruitment of the participants to boost research transferability.

## Results

A total of 36 participants took part in the study. The demographic characteristics of the participants are detailed in Table 1. The participants were divided into seven focus groups and recruited based on age, gender, educational level, position, and job experience. The study observed criteria such as work experience in infectious or Covid-19 departments and obtaining consent to participate (see Table 1).

**Table 1:** Demographic Characteristics of Study Participants

Focus groups	Gender	Age (year)	Job Experience (year)	Educational level
Academic nursing faculty *n* = 6	women	39	13	PhD
	women	42	15	PhD
	men	47	20	PhD
	women	38	10	MSc.
	men	54	28	PhD
	women	50	22	PhD
Clinical nursing faculty *n* = 5	women	33	33	MSc.
	women	60	30	MSc.
	women	37	31	MSc.
	women	40	35	MSc.
	men	41	40	MSc.
Infection control nurses *n* = 3	women	45	15	MD
	men	51	20	MD
	women	41	12	MD
Clinical nursing Supervisors *n* = 5	women	46	23	BSc.
	women	42	18	BSc.
	women	43	18	BSc.
	women	36	10	MSc.
	women	40	15	MSc.
Clinical head nurses *n* = 3	men	40	17	BSc.
	women	38	14	BSc.
	women	40	15	MSc.
Clinical nurses *n* = 5	women	33	9	BSc.
	men	30	8	BSc.
	women	31	6	BSc.
	men	35	10	BSc.
	women	40	15	BSc.
Nursing’s students *n* = 9	men	21	0	BSc. student
	men	22	0	BSc. student
	men	25	0	BSc. student
	men	21	0	BSc. student
	women	21	0	BSc. student
	women	21	0	BSc. student
	women	22	0	BSc. student
	women	24	0	BSc. student
	men	22	0	BSc. student

Note. PhD: Doctor of Philosophy; MD: Doctor of Medicine; BSc: Bachelor of Science; MSc: Master of Science

Finally, 628 codes were extracted from the data analysis of discussions held in 7 focus groups. Three conceptual categories were developed through qualitative analysis: “general characteristics of the curriculum”, “expected competencies and skills”, and “evaluation”. These themes were derived from 9 main categories and 25 subcategories listed in Table 2.

**Table 2:** Extracted Theme, Main, and Subcategories Based on Participants’ Experiences

Theme	Main Categories	Subcategories
General characteristics of the curriculum	Necessity and importance of creating a specialized field	Necessity of creating a specialized field
		Importance of creating a having a specialized field
	Curriculum components	Course Name
		Field Definition
		Mission
		Perspective/Vision
		Course Goal
	teaching method	Educational techniques
		Educational strategies
	Needed educational spaces	Public spaces
		Specialized spaces
	Expected characteristics of graduates	Graduates’ job position
		Graduates’ roles
Expected competencies and skills	Core competencies	Basic skills
		communication skills
	Special competencies	Management
		research
		diagnosis
		educational
		prevention
		care
Evaluation	Learner evaluation	The frequency of evaluation of learners
		Evaluation methods
	Curriculum evaluation	Curriculum revision
		Curriculum standards

### General characteristics of the curriculum

The participants of this research emphasized the vital necessity and importance of obtaining a master’s degree in IPC. This category includes 2 subcategories: “the necessity of creating a field” and “the importance of creating a field”. The participants in the research emphasized the importance of adding the field of IPC to specialized nursing fields. They believe that expanding the fields within nursing would enhance the profession’s level of professionalism. Additionally, an academic staff member expressed their belief in the significance of this expansion. “*The specialization of nursing and the increase of nursing branches will make nursing more professional. Because nursing requires this, how much can a nurse learn in a 4-year bachelor’s course? We need to get out of this shallow sea. Let’s come out.”*

The participants pointed out the need to create a specialized field of IPC due to the lack of engaging content and curriculum, as well as the scattered nature of the topic in undergraduate nursing. Supporting this perspective, one of the head nurses in infectious departments stated: *“I must say that I agree with your opinion. No, it is not enough to teach the subject of the infection in a bachelor’s degree, because infection is a very broad field, that is, it is a multi-dimensional science. He/she must have a strong base, know all the pathogenic agents, know all the hosts that this pathogenic agent can have, know the ways of transmission of the infection, and in the heading of our nursing only in adults, the elderly.”*

The lack of skill and ability of BSc nurses in dealing with infectious diseases in the hospital was highlighted. One participant, with 17 years of experience in clinical nursing management, expressed concern, stating,

*“In the BSc Nursing program, only 0.25 units are dedicated to infectious diseases, providing only basic familiarity with the subject. We do not offer extensive information to the students in this area. Although we have an infection control workshop for undergraduate students as part of the curriculum, we still face significant weaknesses in infection control practices.”*

The feedback from participants on the features of the MSs in preventing and control infection was categorized into five subcategories. Most participants agreed that it was important to include the education level and field in the course name and to emphasize both infection prevention and control. For example, an infectious disease supervisor said,

*“When naming the field, the degree (in this case, a master’s degree) and the specific field (nursing) must be mentioned. We should also pay attention to both prevention and infection control categories in the title, as my colleagues have mentioned.”*

The participants emphasized the importance of having a broad understanding of preventing and controlling infections, specific competencies, professional views, and paying attention to the multidimensionality of infectious science in defining the field. One of the participants highlighted:

“*It’s true, in addition to these, he/she must know professional behaviors such as teamwork, responsibility, and general professional behaviors related to infectious disease specialist nurses.”*

The participants discussed the field’s mission to promote two categories of infection prevention. One participant stated:

*“The mission of this field is to reduce infection and, more importantly, to prevent infection, especially viral and contagious infections. Because these diseases put pressure on societies and people from all points of view, whether social, economic, political, educational, or many other issues. They give as we witnessed.”*

In addition, other participants considered establishing an interdisciplinary connection with the design of this field and broadening nurses’ mental scope on infectious issues as other goals of this field.

The participants emphasized that the curriculum’s perspective was to reduce infection and death rates caused by infectious diseases. An academic member stated:

*“Our vision should be to prevent infectious diseases, and if it happens, we should do the least damage in terms of mortality, economic and social. Not to be paralyzed. We should be able to be the best in the field of infection control in the region and our statistics should be the lowest. This field with educated graduates can help a lot in the future.”*

One of the senior nursing students in the final year said:

*“I agree with my peers’ opinion. The existence of this field can be very effective and can help us prevent the loss of many lives in the face of infectious diseases in the future.”*

The majority of participants mentioned that their primary goals included attention to infection prevention and control as two separate and main goals. Another goal that received significant attention was improving quality and meeting infection control standards. Two participants expressed the following thoughts:

“In broad terms, you have prevention and infection control. These two categories are separate.”

“The overarching goal is to provide quality and standard infectious nursing care to the community.”

The other goals mentioned by participants include increasing sensitivity by becoming more specialized in nursing, developing problem-solving abilities, promoting infection prevention behaviors as a cultural norm, professionally implementing learned practices, providing infrastructure for hygiene and prevention, and increasing responsibility through nursing specialization.

### Expected competencies and skills

In this category, a master of science student needs the required qualifications, competencies, and skills for preventing and controlling infection. According to the participants in the focus group discussion sessions, two sub-categories including “General Capabilities” and “Special Capabilities” were identified.

Core competencies refer to the basic and communication skills for each nurse, including specialist nurses, controlling infection, which is vital according to the views of the participants.

One of the senior year nursing students said:

*“I’m a student in my senior semester now, they give me a ward with twenty patients, and they say all the dressings, I&Os, tests, etc. are with you. How can I do all these things, that’s right? I can’t even take care of myself I will comply, no matter what happens with the patient. In my opinion, it is very important to think of measures and train the nurses themselves so that they can protect themselves against infectious diseases in the first place.”*

One of the nurses in the infectious department added:

“The nurses should receive regular training by participating in workshops, seminars, and infectious disease conferences. This will help them learn new tips in the field of care, education, and other relevant areas.” Deep and effective communication skills can enhance interpersonal relationships. A supervisor stated,

*“One of the skills and responsibilities of an infectious disease specialist nurse, like other nurses, includes observing ethical principles, establishing proper communication with other medical teams, protecting the rights of patients and their families, establishing proper communication with patients and their companions, and students, especially in teaching hospitals. I think this is important.”*

The term “special competences” refers to the skills and qualifications that a specialized nurse must possess to effectively provide services in their field. It encompasses 6 subcategories: “management”, “educational”, “research”, “diagnosis”, “prevention”, and “care”. A clinical faculty member stated,

*“A specialized nurse must have advanced competence and capability, requiring more extensive training and knowledge than a bachelor’s degree, especially in management.”* One of the educational faculty members stated, *“The current infection control supervisors, like many others, have limited knowledge of disease control. For example, if you ask them about infections in more detail, they won’t be able to provide much information.”*

Based on the majority opinion of the participants, a nurse specializing in infection control should possess the necessary competencies and abilities to effectively manage the nursing team with correctness and principles. Additionally, they should enhance their knowledge of advanced management and crisis management, and provide more effective infection prevention and control management.

One head nurse remarked, “Having spent 10 years in the hospital, I’ve observed that current supervisors only possess superficial knowledge.”

One of the infection control nurses said:

*“It certainly has a series of specific duties, of course, we are not too involved in the duties of respected nurses, but in my opinion, this nurse should have specialized competencies in the field of care, such as acquiring skills in various types of culture and tests, correct drug therapy, and timely diagnosis of symptoms in infectious patients. And inform the doctor, know how to change sterile dressings and correctly perform sterile procedures in the correct way, and have specialized information about various infectious diseases.”*

A professional nurse in infection prevention and control should be responsible for educating patients, their companions, and other medical staff. Additionally, community-level education can help in more effective prevention.

“The opinion of one of the nurses in the infectious disease department was: ‘Well, this nurse can have many roles. For example, an educational role is important in infection control and prevention. Duties such as training employees and personnel working in the infectious departments and the ICU, educating patients and their families, and contributing to public education are also crucial.‘”.

One of the ICU head nurses also stated, “The role of the teacher is crucial in this nursing specialty. Proper and basic training can prevent many infectious diseases or expedite treatment.”

One of the supervisors from the Infectious Disease department said, “Yes, we don’t have such facilities at all. We don’t even know. I’ve been at the COVID-19 Center for a year, and I can tell you that if our nurses had more knowledge and awareness, we would have fewer problems.”

### Evaluation

This theme focuses on evaluation and related factors. Based on discussions in the focus groups, two main categories emerged: “learner evaluation” and “curriculum evaluation.” After implementing educational programs, the most important activity is evaluating students. Evaluation is a process used to determine the level of achievement of educational goals for both the teacher and student.

Participants suggested that evaluation should be continuous, not just a final assessment.

One of the clinical faculties said: *“In addition to the final evaluation, student evaluation should also be continuous. Because feedback is needed in order to make the necessary decisions in order to increase the effectiveness of programs or activities.”*

Among the evaluation methods mentioned by the participants and considered appropriate for the evaluation of learners were OSCE, PMP, DOPS, written test and 360-degree test. One of the clinical faculties said: *“To evaluate students in theoretical topics, a written exam is required. For practical units, field assignments and internships are necessary, and in my opinion, practical skills must be evaluated.”*

One of the faculty members said: *“Having a log book is also necessary and it should be known what skills and qualifications our students have acquired.”*

Also, one of the clinical faculties pointed out: *“I think a specialist student should have a portfolio for evaluation, and this is one of the standards of nursing master’s education.”*

The participants emphasized the need to revise the program based on emerging diseases. One of the educational faculties said:

*“Of course, in order to revise the curriculum, you must first conduct a survey, conduct studies, ask the opinions of experts, and then do it, and the curriculum will be revised and updated in this way.”*

Also, one of the nurses mentioned:

*“Because emerging infectious diseases are created and medicines, care, and ways of transmission and protection change, this designed curriculum must be regularly reviewed and evaluated.”*

## Discussion

This qualitative study aims to gather nurses’ viewpoints on the necessity of infection prevention and control (IPC) nurses. The design of a tailored curriculum for nurse training is crucial, given that nurses are key stakeholders and possess a critical function within healthcare settings. This study utilizes the perspectives of nurses to design a curriculum that is fitting and effective. The stakeholders’ point of view is important because involving them in curriculum design can lead to a more comprehensive and localized curriculum, as well as facilitate its implementation. Taking into account the opinions of all stakeholders can help create a more complete and suitable educational program that addresses the weaknesses and shortcomings of nursing knowledge in Iran. In this study, a total of 628 codes were extracted through qualitative content analysis of focus group views. These codes were then organized into nine main categories, 25 subcategories, and three themes, all of which are elaborated upon in this section. In the following, the categories and subcategories extracted in the study are discussed.

The lack of a well-defined, specialized curriculum for infection prevention and control nursing in Iran has resulted in significant gaps and deficiencies within the functions of healthcare and nursing teams. This issue has become particularly apparent during specialist conditions, such as the COVID-19 pandemic, highlighting the urgent need for a comprehensive program in infection prevention. Research participants have noted that these deficiencies were exacerbated during the pandemic. Studies indicate that the lack of specialized nurses had adverse effects on treatment outcomes and patient satisfaction [26, 27]. Jia et al. emphasize the importance of nurses possessing knowledge about infectious diseases to effectively navigate the ethical complexities that arise in nursing practice within this context [28]. As the previous studies indicated a lack of curriculum, participants emphasized the design of the master’s curriculum of infection prevention and control due to the lack of infectious content in the nursing bachelor’s program and the need for specialist nurses to take better care of patients and acknowledged the importance of becoming more specialized in nursing. The curriculum design aimed to bridge the gap between nursing knowledge and clinical practice by providing a comprehensive and accurate view. It also aimed to address the existing lack of curriculum within the country’s educational system to a certain extent. Therefore, according to the general procedure, a course title was designed. The purpose of a course title is to provide a brief overview of the course topic to various audiences. These audiences may include current and prospective students, potential employers, accrediting bodies, other academic institutions, and various other groups on and off campus. The title indicates the level, field, and specialization desired by the graduates of that field [29]. The course title should be concise, specific, and effectively represent the subject matter covered in the course [30]. The conducted studies show, among the infection prevention and control curriculum, only 3 curricula in Korea, Sweden and America were for nurses and were specifically designed for them [31–33].

In this study, the course title was chosen based on participant feedback, consistent with similar studies, and in this study, the title “Master in Infection Prevention and Control Nursing” was considered appropriate by the participants as it includes relevant keywords and effectively represents the focus of the course. The specialization in “infection prevention and control” sets this nursing program apart from others. According to Lewandowski et al., specialized training enables general nurses to concentrate on this specific area, leading to a broader perspective and increased professional effectiveness [34].

Other studies in this field have shown that the presence of infectious disease specialist nurses is crucial for safeguarding mental health, promoting economic development, and maintaining social stability [35, 36].

In clinical settings, preventing and controlling infections requires collaboration among all members of the treatment team, and nurses play a crucial role in this interdisciplinary approach. Stout emphasizes the importance of nursing in infection prevention and highlights the need for collaboration with a multidisciplinary team (MDT) consisting of specialist physicians, microbiologists, pharmacists, and transplant specialists [37]. Some studies highlight the essential role of nurses in infection control and prevention. For example, Wang et al. emphasize the need for specialized nurses with specific qualifications to effectively eliminate infections. They examined the importance of having infection prevention and control nursing as a global standard in every hospital [38]. The research highlighted the crucial role of nurses in infection control and prevention. An essential requirement for specialized infection prevention and control nurse training is the presence of an educational curriculum.

The presence of a comprehensive, organized, and tailored curriculum is crucial for infection control and prevention. According to Ladak et al., countries that incorporate specialized infection prevention and control services into their healthcare teams are more successful in preventing infections and reducing mortality from infectious diseases [39]. Additionally, Alojaimy et al. found that the goal of this curriculum should be to enhance the quality of care for infectious patients and to meet the necessary standards for infection control while addressing cultural considerations [40].

The curriculum is important, but there are different ways to teach infection prevention and control principles. While curriculums are commonly used in educational institutions, nursing staff can also learn through self-service training in clinical settings. There are many strategies for teaching. Participants in the focus group discussion mentioned strategies such as student-centered, project-based, and evidence-based. In their study, Miraglia et al. found that it is more effective to be student-centered in higher education, and because of the student’s effort in understanding scientific topics, meaningful learning is created [41]. Breytenbach et al. believe in the benefits of adopting an evidence-based approach in nursing that is useful to policy makers. They can gain more knowledge about issues that lead to more rational decision-making [42].

Another the methods mentioned by the participants for educating students in this field were question and answer, discussion and using problem solving methods in education. Zhao et al. found that the ability and skill of problem-solving help people to solve the problems that have occurred in their lives; Use these challenges as an opportunity to flourish their talents and have a creative and idea-generating mind, and this method is very effective for education [43].

In Iran, there is a strong need for a structured curriculum to prevent and control infections, especially in critical situations such as the coronavirus pandemic. Currently, infection prevention and control are taught in a general manner in textbooks, and the per-bed teaching method is used to teach these principles in clinical settings [44, 45]. Participants pointed to the lack of nurses, especially during the epidemic of infectious diseases such as Covid. They stated that there was no sufficient and specialized information for controlling infection in the nursing team and they experienced many problems in the field of nursing due to the lack of IPC specialist nurses.

According to the participants, suitable workplaces for an IPC specialist nurse are: public and private hospitals, health centers and clinics, schools, nursing homes, student dormitories, barracks, wound clinics, centers consulting and providing nursing care at home, research centers and institutes related to infection prevention and control, educational centers in universities and hospitals. For a specialist nurse who has learned skills and specialized science in a specific field of nursing, for more effective results, it is better to provide services in the role and workplace related to his specialty [46]. Although most common in the hospital, nurses may provide services in other settings [47].

Findings showed that the participants considered IPC nurses as a necessity to communicate with the client, companions, nursing team and other health teams. Among the health care providers, the only group that has a direct and long relationship with the client is the nursing group [48]. Tuohy considers communication as the most basic ability and skill of nursing [49]. Many studies show that by increasing communication skills, patient satisfaction and the quality of nursing services increase [50, 51].

Basic skills such as self-promotion and professional accountability, mastering problem-solving skills and providing needed solutions, focusing on self-safety, developing responsibility and accountability, and protecting the rights of clients that the participants in this research mentioned. It is necessary not only for IPC specialist nurses but also for all nurses. Appropriate responsibility and accountability for the actions taken by the nurse, increases the quality of care and patient satisfaction [52].

Special competencies are actually having special skills for an IPC nurse to perform the assigned specialized tasks with higher quality. These duties can include: education, research, prevention, management, diagnostic and care fields.

Evaluation is very important in education as quality education and promoting educational programs is the main concern of the entire educational system [53]. Curriculum evaluation is done in order to judge or agree on the value of a curriculum. Therefore, the main role of evaluation is to determine the value of the curriculum. In sum, there is a need to integrate competency assessment methods into skills and competence that are providing nursing care to customers [54].

## Conclusions

In the opinion of the stakeholders the presence of IPC can help to diagnosis, effective care and education of infectious patients to reduce morbidity and mortality. Weakness in proper management of the infectious team and treatment departments, correct monitoring of infection control guidelines and Crisis management are among the shortcomings of the nursing team in this field in Iran, which the participants believed that with the addition of IPC specialist nurses, these duties will be covered. Therefore, the presence of infectious disease specialist nurses is vital. The emergence of newly emerging diseases at the global level and the establishment of a specialized field of infection prevention and control should be considered by nursing education planners. The presence of an infectious disease specialist nurse with academic education can be effective in the prevention and control of chronic, hospital-acquired and emerging infectious diseases in developing countries. It is also suggested that other policy makers and decision makers in the nursing field of other less developed countries should put this issue on their agenda.

### Strength and limitations

The strength of this study is the maximum diversity of participants in age, educational level, years of experience, and various roles. The limitation of the study is that the face-to-face interaction was less due to the busyness of the participants, they participated in the discussions virtually.

## Data Availability

The datasets used the current study are available from the corresponding author on reasonable request.

## Abbreviations

IPC: Infection Prevention Control
WHO: World Health Organization

## References

[1] Bloom DE, Cadarette D, 1 (2019). Infectious disease threats in the twenty-first century: strengthening the global response. Front Immunol.

[2] Becker K, Hu Y, Biller-Andorno N, 2 (2006). Infectious diseases - a global challenge. Int J Med Microbiol.

[3] Turale S, Meechamnan C, Kunaviktikul W, 3 (2020). Challenging times: Ethics, nursing, and the COVID-19 pandemic. Int Nurs Rev.

[4] Amer HA, Alowidah IA, Bugtai C, Soule BM, Memish ZA (2022). Challenges to the infection control team during coronavirus disease 2019 (COVID-19) pandemic in a quaternary-care medical center in Saudi Arabia. Infect Control Hosp Epidemiol.

[5] Kraft M, Kästel A, Eriksson H, Hedman AR, 5 (2017). Global nursing a literature review in the field of education and practice. Nurs Open.

[6] Galanis P, Vraka I, Fragkou D, Bilali A, Kaitelidou D (2021). Nurses’ burnout and associated risk factors during the COVID-19 pandemic: a systematic review and meta‐analysis. J Adv Nurs.

[7] Fulton JS, Mayo A, Walker J, Urden LD (2019). Description of work processes used by clinical nurse specialists to improve patient outcomes. Nurs Outlook.

[8] Mayo AM, Ray MM, Chamblee TB, Urden LD, Moody R (2017). The Advanced Practice Clinical Nurse specialist. Nurs Adm Q.

[9] Kerr H, Donovan M, McSorley O (2021). Evaluation of the role of the clinical nurse specialist in cancer care: an integrative literature review. Eur J Cancer Care (Engl).

[10] Sanchez KMSN, CCRN-K RN, Winnie ACCNS-AG, Kathrine DNP, CCRN-K RN, de Haas-Rowland AGCNS-BC, Natalie MSN, RN, CCNS-CCRN-CSC-CMC. Establishing the Clinical Nurse Specialist Identity by Transforming Structures, Processes, and Outcomes. Clinical Nurse Specialist 33(3): p 117–122, 5/6 2019. | 10.1097/NUR.0000000000000438. 10.1097/NUR.0000000000000438 30946108

[11] Cooper MA, McDowell J, Raeside L, ANP, –, CNS Group (2019). The similarities and differences between advanced nurse practitioners and clinical nurse specialists. Br J Nurs.

[12] Fischer-Cartlidge E, Short K (2023). Successful clinical nurse specialist recruitment: creating a Talent Pipeline. Clin Nurse Spec.

[13] Lee MH, Jun SH (2022). Factors affecting the Infection Control Practices of Nurses at University Hospitals. Healthc (Basel).

[14] Dawson SJ (2003). The role of the infection control link nurse. J Hosp Infect.

[15] Massaroli A, Martini JG, Moya JLM, Pereira MS, Tipple AFV, Maestri E (2019). Skills for generalist and specialist nurses working in the prevention and control of infections in Brazil. Rev Lat Am Enfermagem.

[16] Wu C, Yan J, Wu J, Wu P, Cheng F, Du L (2021). Development, reliability and validity of infectious disease specialist nurse’s core competence scale. BMC Nurs.

[17] Kim KM, Jeong JS, Park HR (2010). Infection control nurse specialist education in Korea. Am J Infect Control.

[18] Gail C, Field KW, Simpson T, Bond EF (2004). Clinical nurse specialists and nurse practitioners: complementary roles for infectious disease and infection control. Am J Infect Control.

[19] Halcomb E, McInnes S, Williams A, Ashley C, James S, Fernandez R (2020). The experiences of primary healthcare nurses during the COVID-19 pandemic in Australia. J Nurs Scholarsh.

[20] Burnett E (2018). Effective infection prevention and control: the nurse’s role. Nurs Stand.

[21] Heinen M, van Oostveen C, Peters J, Vermeulen H, Huis A (2019). An integrative review of leadership competencies and attributes in advanced nursing practice. J Adv Nurs.

[22] Wang J, Liu F, Zhou M, Lee YF (2020). Will the status of infection prevention and control (IPC) professionals be improved in the context of COVID-19?. Am J Infec Control.

[23] Gustafson T (2006). Three uses of the standardized infection ratio (SIR) in infection control. Infect Control Hosp Epidemiol.

[24] Turale S, Meechamnan C, Kunaviktikul W (2020). Challenging times: ethics, nursing, and the COVID-19 pandemic. Int Nurs Rev.

[25] Gunawan J (2015). Ensuring trustworthiness in qualitative research. Belitung Nurs J.

[26] Vejdani M, Foji S, Jamili S, Salehabadi R, Adel A, Ebnehoseini Z (2021). Challenges faced by nurses while caring for COVID-19 patients: a qualitative study. J Educ Health Promot.

[27] Lopatina E, Donald F, DiCenso A, Martin-Misener R, Kilpatrick K, Bryant-Lukosius D (2017). Economic evaluation of nurse practitioner and clinical nurse specialist roles: a methodological review. Int J Nurs Stud.

[28] Jia Y, Chen O, Xiao Z, Xiao J, Bian J, Jia H (2021). Nurses’ ethical challenges caring for people with COVID-19: a qualitative study. Nurs Ethics.

[29] Muraraneza C, Mtshali NG, Mukamana D (2017). Issues and challenges of curriculum reform to competency-based curricula in Africa: a meta‐synthesis. Nurs Health Sci.

[30] Maddalena V, Pendergast A, McGrath G (2018). Quality improvement in curriculum development. Leadersh Health Serv (Bradf Engl).

[31] College of Medicine [Internet]. The Catholic University of Korea [cited 2022 Dec 01]. https://medicine.catholic.ac.kr/eng/.

[32] Swedish red cross university [Internet]. Programs and courses [cited 2022 Dec 01]. https://www.rkh.se/en/programmes-and-courses-at-the-swedish-red-cross-university-college//.

[33] Post University [Internet]. Master of Science in Nursing – Infection Prevention and Control Specialization [cited 2022 Dec 01]. https://post.edu/academics/online-graduate-degrees/m-s-nursing/infection-prevention-and-control/.

[34] Lewandowski W, Adamle K (2009). Substantive areas of clinical nurse specialist practice: a comprehensive review of the literature. Clin Nurse Spec.

[35] Lee HJ, Kim DH, Na YJ, Kwon MR, Yoon HJ, Lee WJ (2019). Factors associated with HIV/AIDS-related stigma and discrimination by medical professionals in Korea: a survey of infectious disease specialists in Korea. Niger J Clin Pract.

[36] Benzian H, Greenspan JS, Barrow J, Hutter JW, Loomer PM, Stauf N (2015). A competency matrix for global oral health. J Dent Educ.

[37] Staudt MD, The Multidisciplinary Team in Pain Management (2022). Neurosurg Clin N Am.

[38] Wang L, Qi N, Zhou Y, Zhang H (2020). Prevention and infection control of COVID-19 in nursing homes: experience from China. Age Ageing.

[39] Ladak A, Lee B, Sasinski J (2021). Clinical nurse specialist expands to crisis management role during COVID-19 pandemic. Clin Nurse Spec.

[40] Alojaimy RS, Nakamura K, Al-Sobaihi S, Tashiro Y, Watanabe N, Seino K (2021). Infection prevention and control standards and associated factors: Case study of the level of knowledge and practices among nurses in a Saudi Arabian hospital. J Prev Med Hyg.

[41] Miraglia R, Asselin ME (2015). Reflection as an educational strategy in nursing professional development: an integrative review. J Nurses Prof Dev.

[42] Breytenbach C, Ten Ham-Baloyi W, Jordan PJ (2017). An Integrative Literature Review of evidence-based teaching strategies for nurse educators. Nurs Educ Perspect.

[43] Zhao W, He L, Deng W, Zhu J, Su A, Zhang Y (2020). The effectiveness of the combined problem-based learning (PBL) and case-based learning (CBL) teaching method in the clinical practical teaching of thyroid disease. BMC Med Educ.

[44] Ahmadi Chenari H, Zakerimoghadam M, Baumann SL (2020). Nursing in Iran: issues and challenges. Nurs Sci Q.

[45] Fathabad HS, Yazdanpanah A, Hessam S, Chimeh EE, Aghlmand S (2015). Organizational Justice and the shortage of nurses in Medical & Educational hospitals, in Urmia-2014. Glob J Health Sci.

[46] Mohr LD, Coke LA (2018). Distinguishing the clinical nurse specialist from other graduate nursing roles. Clin Nurse Spec.

[47] Lu H, Zhao Y, While A (2019). Job satisfaction among hospital nurses: a literature review. Int J Stud Nurs.

[48] Al-Kalaldeh M, Amro N, Qtait M, Alwawi A. Barriers to effective nurse-patient communication in the emergency department. Emerg Nurse. 2022;30(5). 10.7748/en.2020.e1969. 10.7748/en.2020.e1969 32285654

[49] Tuohy D (2019). Effective intercultural communication in nursing. Nurs Stand.

[50] Lotfi M, Zamanzadeh V, Valizadeh L, Khajehgoodari M (2019). Assessment of nurse–patient communication and patient satisfaction from nursing care. Nurs Open.

[51] Burgener AM (2020). Enhancing communication to improve patient safety and to increase patient satisfaction. Health Care Manag (Frederick).

[52] Christensen SS (2019). Escape from the diffusion of responsibility: a review and guide for nurses. J Nurs Manag.

[53] Kim HS (2012). Outcomes-based curriculum development and student evaluation in nursing education. J Korean Acad Nurs.

[54] Ruhe V, Boudreau JD (2013). The 2011 program evaluation standards: a framework for quality in medical education programme evaluations. J Eval Clin Pract.

